# Gleanings in Science and Medicine

**Published:** 1893-05-27

**Authors:** 


### Gleanings in Science and Medicine.

March was a very fatal month in New York. Returns show that the number of deaths exceeded the average of 2,000. Influenza is held responsible for a large proportion of this mortality, and deaths from acute respiratory diseases were in preponderance.

A very sensible Bill has been brought before the Legislature of Pennsylvania, which would prohibit the exhibition of monstrosities in public places. The morbid taste for horrors is not one that civilisation should encourage. It was a good day when executions were no longer made public spectacles, and no harm would be done if the exhibition of monstrosities at fairs and the like were prohibited also.

A French physician, Dr. Mesnet, has made a series of observations undertaking to prove that somnambulism is a result of hysteria, and that ecstasy, catalepsy, and lethargy are all phases of the same condition. Practical observations—and these are backed up by medical research—point rather to an imperfect process of nutrition. An ill-balanced condition of nerves is frequently noticeable in persons given to somnambulism, but this by no means need imply hysteria.

Professor Bayer, of Munich, who has been making researches in some of the most ancient tombs of Achmin, has come upon some unlooked-for results among the mummies of certain Egyptian Princesses lying there. These discoveries are no less than certain vials containing cosmetics and beautifying lotions by the side of their dead and gone mistresses, from whom these precious belongings were not separated even in death. But whilst the lady died, and her beauty departed, the lotions which beautified her lived on, and Professor Bayer is now making an analysis of the contents of the precious vials, the results of which are to be published for the benefit of the feminine world of to-day.

The course of lectures which Professor H. Drummond has been giving in the United States, have attracted large audiences. The line of argument in these discourses, if carried far enough, is suggestive of man as a disembodied metaphysician than as the metaphysical animal into which the march of human intellect is fast transforming him. The author of "Natural Law in the Spiritual World" has pointed to the decadence of athleticism around us—a decadence, fortunately, which we fail to observe with the lecturer. Indeed, it seems to us rather as if the trip across the Atlantic had obliterated from Professor Drummond's mind the memory of the cricket fields and the rivers of his native shores; whereon, now, as formerly, the reign of the muscle is supreme.

Edinburgh has ever shown much kindly toleration to female aspirants of medical practice, it was, in fact, to the Scottish capital that the lady doctors of to-day owed indeed their professional existence. We shall now be anxious to see if the gallantry of the past will be further extended in the future. A certain lady practitioner has recently applied for admittance to one of the great medical societies of the University. There was no rule made when this society was first founded against the admittance of members of the female sex, and it will be interesting to know what the ultimate decision of the committee will be regarding the request.

It is little to be wondered at that the streets in London are so well kept, if we bear in mind how much labour is employed over this work. In the City itself, of which part of the metropolis we are now speaking, the 480 miles of streets, which are comprised in one square mile, are continually under the charge of 500 scavengers, whilst work also in the day time is found for some 150 boys. Saturday night is entirely spent by this brigade from midnight until nine o'clock on Sunday morning cleansing and sweeping the thoroughfares. The actual water used yearly n this work is as much as 4,000,000 gallons.

It is always difficult to separate customs from their abuses, and in our judging of them we are apt to let our minds be over-biased by the carrying to excess of what may be in itself a harmless thing. And so it certainly is with regard to the opium question in India. With the bare mention of the drug our mind flies to such haunts as have received graphic descriptions at the hands of more than one popular novelist of the day. In India, indeed, opium dens exist less in fact than in fiction; English agitators in this respect have let their philanthropy carry them away in a curiously exaggerated manner. Opium smoking and opium dens only have the same relation to one another that the ordinary consumer of a daily glass of claret at dinner bears to the habitual lounger in a public-house. As taken in India opium is not indulged in in any large quantities, neither is it so harmful as our every day alcoholic stimulants are to us. Indeed, Sir George Birdwood affirms that the evil of the Indian habit is less injurious than that of our European one; whilst, on the same authority, we are told that opium is actually beneficial to the native population; it is a certain and effectual preventive to malarial fever, and stops all craving for stimulants. The Chinese habit cannot be defended in the same manner for its moderation as the Indian can; but there, again, it is the abuse and not the thing itself which is at fault.

